# Enhancement Strategies in Transition Metal Oxides as Efficient Electrocatalysts for the Oxygen Evolution Reaction

**DOI:** 10.3390/molecules31010147

**Published:** 2026-01-01

**Authors:** Pengxin Li, Ning Song, Naxiang Wang, Yan He, Zhi Zhu, Yongsheng Yan

**Affiliations:** School of Chemistry and Chemical Engineering, Jiangsu University, Zhenjiang 212013, China

**Keywords:** TMOs, electrocatalysts, OER, enhancement strategies

## Abstract

Hydrogen energy has been recognized as the most promising secondary energy source due to high energy density, abundance, and environmental friendliness. Among hydrogen production techniques, water electrolysis has emerged as a key research focus, owing to its high efficiency, operational simplicity, controllability, and pollution-free nature. However, the anodic oxygen evolution reaction (OER) involves a high overpotential and sluggish kinetics, which severely constrain the overall efficiency of water electrolysis. Transition metal oxide (TMO) catalysts are regarded as promising substitutes for noble-metal-based catalysts, given their advantages of low cost, elemental abundance, tunable electronic structures, and favorable stability. This review systematically elaborates on the reaction mechanisms of TMO catalysts, including the adsorbate evolution mechanism (AEM) and lattice oxygen mechanism (LOM), and summarizes various performance-enhancement strategies, such as morphology control, doping engineering, support engineering, and heterostructure construction. Furthermore, it outlines current challenges and future research directions, covering precise synthesis and structural control, identification of active sites and mechanistic elucidation, and stability and degradation issues, as well as multifunctional applications and broad-pH-range adaptability. The aim is to offer theoretical guidance and technical insights for designing and developing high-performance TMO electrocatalysts.

## 1. Introduction

With the accelerated global energy transition, the development of efficient and clean energy conversion and storage technologies has risen to the forefront of scientific research [1,2,3]. As a green energy source, hydrogen energy has acquired tremendous attention due to high calorific value, zero-emission characteristics, and high specific energy density [4,5]. Among various hydrogen production methods, water electrolysis stands out as a particularly economical and environmentally friendly option, especially suitable for integration with renewable energy sources [6,7]. The water electrolysis process comprises two half-reactions: the cathodic hydrogen evolution reaction (HER) and the anodic oxygen evolution reaction (OER) [8]. The OER is a complex four-electron transfer process characterized by sluggish reaction kinetics and a requirement for high overpotential, making it the critical factor limiting the overall energy efficiency of water electrolysis [9,10]. Currently, commercial electrocatalysts typically employ noble metal-based catalysts (such as IrO_2_ and RuO_2_) to lower the OER overpotential. However, the high cost and insufficient stability of noble metal catalysts severely hinder their large-scale application [11,12,13,14]. Consequently, the development of highly efficient, low-cost, and durable non-precious metal OER electrocatalysts has become a shared objective of both the scientific research community and industry.

In recent years, non-precious metals (such as Mn, Co, Ni, and Fe) and their compounds, including oxides, sulfides, phosphides, nitrides, and hydroxides, have been widely investigated as heterogeneous catalysts. These materials exhibit promising multifunctional electrocatalytic performance, especially in the OER [15,16,17,18]. A key advantage of these catalysts is the significantly lower cost compared to noble-metal-based counterparts while still retaining high catalytic activity and sufficient durability, even under harsh oxidizing conditions [19,20,21]. These attributes make them highly suitable for water-splitting applications. Among the aforementioned materials, transition metal oxides (TMOs), particularly those based on Fe, Co, Ni, Mn, and their composite oxides, show great application potential for OER [22,23]. These materials are not only earth-abundant and low-cost but can also achieve electrocatalytic property comparable or even superior to precious metal catalysts through rational composition design and structural modulation [24,25,26,27]. Nonetheless, TMO catalysts still face numerous challenges in practical applications, primarily including limited intrinsic activity, poor electrical conductivity, and structural instability under harsh OER conditions [28,29]. To overcome these bottlenecks, researchers have developed various enhancement strategies, optimizing catalyst design from multiple dimensions, such as electronic structure, nanostructure, and interface engineering [30,31,32,33]. In recent years, with the fast development of advanced preparation methods and detecting techniques, especially in situ characterization techniques and theoretical simulations, deeper understanding has been gained regarding active site identification, reaction mechanisms, and structure–property relationships during the OER process [34,35,36,37].

This paper summarizes the recent advances in performance enhancement strategies for TMO-based OER electrocatalysts. It will focus on discussing the OER mechanisms, the application of advanced synthesis and characterization techniques in catalyst research, and propose future development directions addressing current challenges. The goal is to afford theoretical and technical insights for the design and development of high-performance TMO electrocatalysts.

## 2. Reaction Mechanisms and Performance Regulation

### 2.1. Fundamental Mechanisms of the OER

The OER proceeds primarily through two competing mechanisms: the adsorbate evolution mechanism (AEM) and the lattice oxygen mechanism (LOM) [38,39]. The traditional AEM involves four single electron–proton transfer steps, with O-O bond formation occurring via nucleophilic attack, leading to scaling relationships between the adsorption energies of intermediates and a theoretical lower limit for the overpotential of 0.37 V [40,41]. In contrast, the LOM involves direct participation of lattice oxygen in O-O bond formation, breaking the scaling relationship restrictions and potentially allowing for further reduction of the reaction overpotential [42,43].

**Figure 1:**
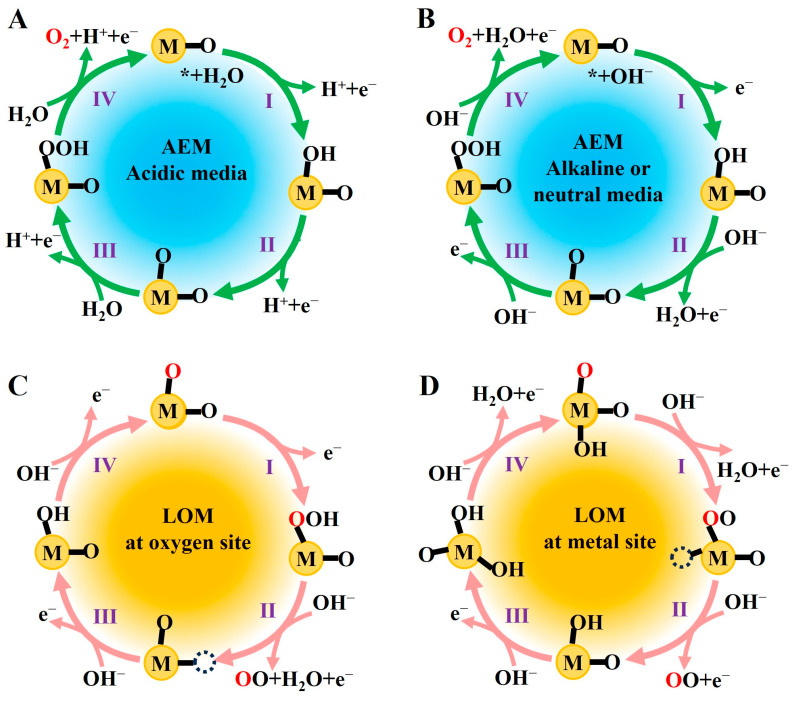
Schematics of OER mechanisms: AEM pathways under (**A**) acidic and (**B**) alkaline/neutral conditions; LOM pathways on (**C**) the oxygen site and (**D**) the metal site.

The AEM is the most extensively studied OER pathway (Figure 1A,B), with typical steps including the following [44]:

(1) H_2_O dissociation adsorption: H_2_O adsorbs on the electrode surface and dissociates into H^+^ and OH^−^.
(2) Deprotonation of OH*: OH^−^ undergoes further deprotonation, generating O* and H^+^.
(3) Bond formation: O* combines with another O* to form an O-O bond, producing O-OH*.
(4) Deprotonation of O-OH*: O-OH* loses a proton, generating O_2_ and H^+^.

In the AEM pathway, the formation of O-OH* is the rate-determining step, with a high energy barrier leading to a high overpotential for the OER.

Unlike AEM, the core of LOM lies in directly utilizing oxygen atoms from the catalyst lattice to form oxygen molecules (Figure 1C,D). The fundamental reaction steps include the following [45]:

(1) Lattice oxygen activation: Under applied potential, the covalency of the metal–oxygen bond (M-O) strengthens, and oxygen ligands lose electrons to form electrophilic oxygen species.
(2) Nucleophilic attack: OH^−^ or H_2_O molecules from the electrolyte attack the activated lattice oxygen, forming a superoxide-like group (O^2−^).
(3) Oxygen vacancy formation: The release of lattice oxygen leads to the generation of surface oxygen vacancies (O_v_).
(4) Vacancy repair: Oxygen sources (H_2_O/OH^−^) from the solution fill the oxygen vacancies, restoring the catalyst structure.

The LOM can break the scaling relationship limitations of the AEM, significantly accelerating reaction kinetics, especially showing clear advantages under high-current density conditions.

### 2.2. Performance Regulation of TMOs

The catalytic performance of TMOs is closely related to their electronic structure, particularly the occupancy of 3d orbitals and the position of the d-band center [46,47]. By modulating the d-band center, the adsorption energy of reaction intermediates can be optimized, and the reaction energy barrier can be reduced [48,49]. For example, in F-doped Co_2_NiO_4_, F-doping regulates the d-band center and improves the occupancy of Co and Ni d-orbitals and thereby improves the inherent OER activity [50].

Furthermore, spin-state regulation is also an important influence factor of electrocatalytic activity [51]. For instance, Co^3+^ ions in an octahedral field can be in high-spin (HS t_2g_^4^e_g_^2^), intermediate-spin (IS t_2g_^5^e_g_^1^), or low-spin (LS t_2g_^6^e_g_^0^) states, with different spin states significantly influencing the adsorption strength of intermediates [52]. Elemental doping or interface engineering can regulate the spin state of metal ions, thereby optimizing catalytic performance [53,54].

## 3. Optimization Strategies for the OER Catalytic Performance of TMOs

### 3.1. Morphology Control

The composition and morphological structure of electrocatalysts are key factors for improving the quantity of active sites and increasing the inherent activity of each active site. For OER electrocatalysts based on TMOs, morphology engineering can expand the specific surface area of the catalyst and expose accessible active sites to reactants [55,56,57]. Notably, the design via oriented crystal exposure and architecting multi-dimensional structures is in favor of increasing the density of active sites and facilitating electron/charge transfer, thereby enhancing the comprehensive activity and durability of OER [58,59].

#### 3.1.1. Crystal Orientation Modulation

**Figure 2:**
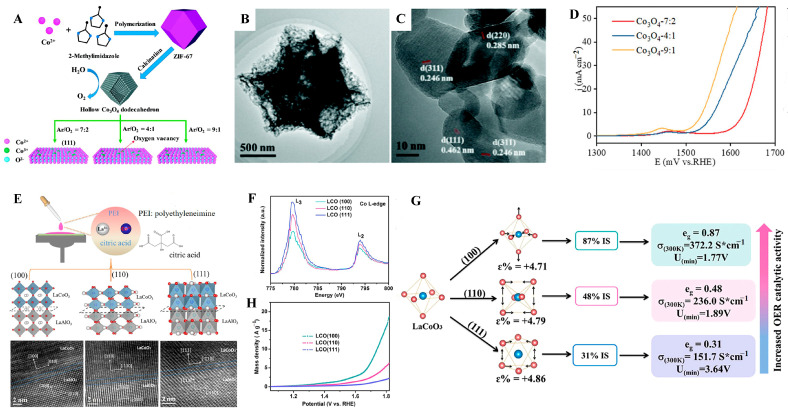
(**A**) Synthesis procedure of hollow Co_3_O_4_ and oxygen content-controlled dominant exposure of the (111) facet. (**B**) TEM and (**C**) HRTEM images of Co_3_O_4_-9:1. (**D**) LSV curves of Co_3_O_4_-9:1, Co_3_O_4_-4:1, and Co_3_O_4_-7:2. Reproduced with permission from Ref. [63]. (**E**) Schematic illustration and HRTEM images, (**F**) Co L-edge and LSV curves, and (**G**) the relationship between OER activity and spin configuration of LaCoO_3_ (100), (110), and (111) films. (**H**) The OER polarization curves of the LCO (100), (110), and (111) films. Reproduced with permission from Ref. [64].

Different crystal faces exhibit distinct thermodynamic and kinetic properties. Therefore, by selectively exposing specific crystal facets, the regulation of crystal orientation can be achieved, thereby optimizing the catalytic performance of TMOs for OER [60,61,62]. Yang et al. [63] used ZIF-67 as the template and adjusted the atmosphere of the thermal treatment process to synthesize a series of hollow Co_3_O_4_ dodecahedra with controllable crystal orientations (Figure 2A,C). These hollow Co_3_O_4_ dodecahedra feature controllable (111) crystal plane exposure and exhibit remarkable OER properties, with an overpotential of 307 mV at 10 mA cm^−2^ and a Tafel slope as low as 55 mV dec^−1^ (Figure 2D). These performances are obviously superior to those of the Co_3_O_4_ on the non-exposed (111) crystal planes. Moreover, Tong et al. [64] proposed a unique spin-state regulation concept to enhance the OER activity of LaCoO_3_ electrocatalysts (Figure 2E). X-ray absorption spectroscopy (XAS) measurements confirmed that the spin state in LaCoO_3_ thin films with different crystal plane orientations transitions from a nearly LS t_2g_^6^e_g_^0^ (e_g_ = 0.31) to an IS t_2g_^5^e_g_^1^ (e_g_ = 0.87) (Figure 2F). The LaCoO_3_ (100) film exhibits the highest electron concentration (up to 0.87) and lower adsorption energy for oxygen intermediates (Figure 2G), thereby demonstrating the optimal OER performance. At an overpotential of 470 mV, its mass activity is 2.9 times and 6.7 times higher than those of the LaCoO_3_ (110) and LaCoO_3_ (111) films, respectively (Figure 2H).

#### 3.1.2. Architecting Multi-Dimensional Structures

**Figure 3:**
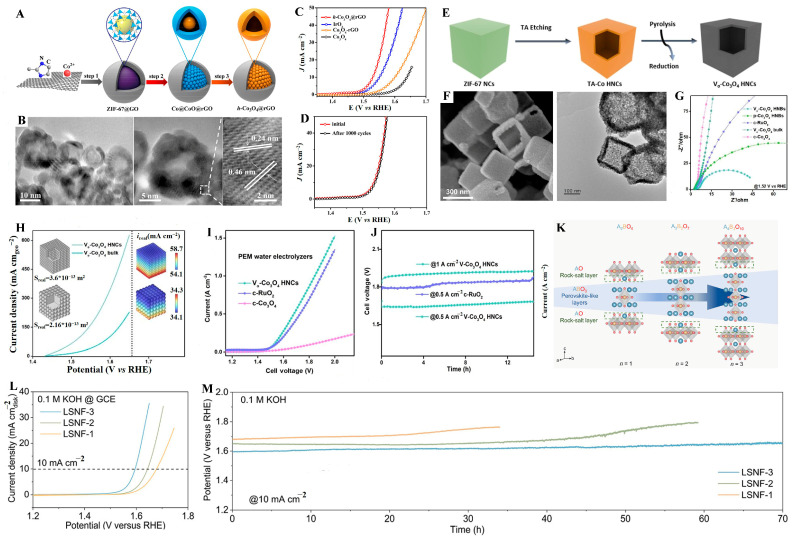
(**A**) Synthesis procedure of the h-Co_3_O_4_@rGO hollow composite and (**B**) HRTEM images of h-Co_3_O_4_@rGO. (**C**) LSV curves of h-Co_3_O_4_@rGO and contrast materials. (**D**) The continuous CV performance of h-Co_3_O_4_@rGO at 5 mV s^−1^ towards OER. Reproduced with permission from Ref. [69]. (**E**) Schematic illustration of the preparation of (**F**) SEM and TEM of the Vo-Co_3_O_4_ HNCs. (**G**) Nyquist plots of the V_o_-Co_3_O_4_ HNCs and contrast materials. (**H**) Finite-element simulations. (**I**) Steady-state polarization curve of PEM electrolyzer using V_o_-Co_3_O_4_ HNCs as anodic catalysts. (**J**) Chronopotentiometry tests at 1 A cm^−2^ in the PEM electrolyzer. Reproduced with permission from Ref. [70]. (**K**) Schematic diagram of a tetragonal crystal structure for RP-type perovskites, A*_n_*_+1_B*_n_*O_3*n*+1_ (*n*  =  1, 2, 3). (**L**) LSV curves. (**M**) Chronopotentiometric curves of synthesized oxides at 10 mA cm^−2^. Reproduced with permission from Ref. [71].

Architecting multi-dimensional structures is a valuable approach for increasing OER performance by modifying the physical morphology of catalysts. It accelerates the reaction rate and boosts the activity of catalysts by increasing the contact area between catalysts and reactants. Its approach involves fabricating various morphologies (such as 2D nanosheets, hollow architecture, and porous structures) to enhance the specific surface area of catalysts and expose more active sites to reactants [65,66,67]. Ahmed et al. [68] obtained CuCo_2_O_4_ catalysts with different morphologies (two-dimensional nanosheets, cubes, and spheres aggregated by particles) via hydrothermal reactions using different solvents. The electrochemical OER performance was enhanced by optimizing the surface structure and size of the two-dimensional CuCo_2_O_4_ nanosheets, while their chemical composition and crystallinity remained unchanged. The optimized two-dimensional CuCo_2_O_4_ nanosheet exhibited excellent electrochemical performance for OER. At a current density of 10 mA cm^−2^, it had an overpotential of 290 mV and showed outstanding long-term stability. Hollow Co_3_O_4_ nanoparticles (h-Co_3_O_4_@rGO) were prepared by Men et al. [69] through annealing the ZIF-67@GO precursor. The enhanced OER catalytic performance of h-Co_3_O_4_@rGO was ascribed to the large specific surface area and unique hollow morphology (Figure 3A,B). Consequently, it showed a low overpotential of 300 mV at 10 mA cm^−2^ and outstanding durability, outperforming the solid Co_3_O_4_@rGO composite (Figure 3C,D).

In addition, morphology engineering can optimize the electronic structure and augment the local electric field intensity, thereby improving the charge/electron transfer capability and OER kinetics. For example, vacancy-rich Co_3_O_4_ hollow nanocubes (V_o_-Co_3_O_4_ HNCs) were formed via the tannic acid etching of ZIF-67, followed by high-temperature calcination in air (Figure 3E,F). Compared with bulk V_o_-Co_3_O_4_, V_o_-Co_3_O_4_ HNCs have a higher specific surface area (303 m^2^ g^−1^ vs. 212 m^2^ g^−1^), which is conducive to the exposure of more active sites and rapid charge/electron/mass transfer (Figure 3G). Finite-element simulation (Figure 3H) results show that the electric field intensity around Co_3_O_4_ HNCs is 7.94 × 10^3^ V m^−1^, nearly twice as high as that of bulk Co_3_O_4_. The higher electric field intensity enhances the E_ads_ of water and electron transport capability, which directly facilitates the kinetic process of OER. Moreover, at different potentials, Co_3_O_4_ HNCs can deliver a higher current density than bulk Co_3_O_4_, which is attributed to its hollow structure that promotes the diffusion of electrolytes into the interior and the reaction on fully exposed active sites. Therefore, the V_o_-Co_3_O_4_ HNCs catalyst was further applied to the OER in PEM. Figure 3I shows that the water electrolyzer with V_o_-Co_3_O_4_ HNC catalysts exhibits extremely high activity. A large current density of 1 A cm^−2^ can be achieved at a cell voltage of 1.82 V (Figure 3J) [70].

Besides the morphology dimensionalities, the molecular crystal dimensionalities are also important for the OER performance. For example, Zhang et al. [71] synthesized a series of Ruddlesden-Popper (RP)-type perovskites (La_0.125_Sr_0.875_)*_n_*_+1_(Ni_0.25_Fe_0.75_)*_n_*O_3*n*+1_(*n* = 1, 2, 3) (denoted as LSNF-1, LSNF-2, and LSNF-3) with tunable crystal dimensionalities, aiming to investigate the correlation between OER activity and stability under different dimensionalities (Figure 3K). It has been demonstrated that materials with a higher dimensionality (n value increase) exhibit faster reaction kinetics, a larger electrochemical active area, and superior electron transport capability (induced by enhanced Ni/Fe-O). Consequently, LSNF-3, which has the highest crystal dimensionality, exhibits higher activity and stability for OER (Figure 3L,M).

### 3.2. Crystal Phase Change

#### 3.2.1. Amorphization

Amorphous materials, a special category of solid materials, exhibit long-range disorder in atomic arrangement and only short-range order over a few atomic distances. Compared with crystalline materials, the amorphous materials contain numerous defects that generate ample dangling bonds, acting as active sites for OER. The long-range disordered atomic packing enables amorphous nanomaterials with loose stacking features and extended bond lengths, resulting in a high surface area at the atomic scale. Therefore, amorphous materials frequently outperform their crystalline counterparts in electrocatalytic applications, owing to distinctive structural attributes, such as good structural flexibility, tunable chemical composition, and plentiful active sites [72,73]. Duan et al. [74] successfully synthesized amorphous NiFeMo oxides with homogeneous elemental distribution using a simple co-precipitation method. Compared with their crystalline counterparts, the amorphous NiFeMo oxides underwent a more rapid surface self-reconstruction in the OER process, forming a metal-oxy(hydroxide) active layer with rich oxygen vacancies. This structural evolution resulted in exceptional OER activity, achieving an overpotential of 280 mV at 10 mA cm^−2^ in 0.1 M KOH.

**Figure 4:**
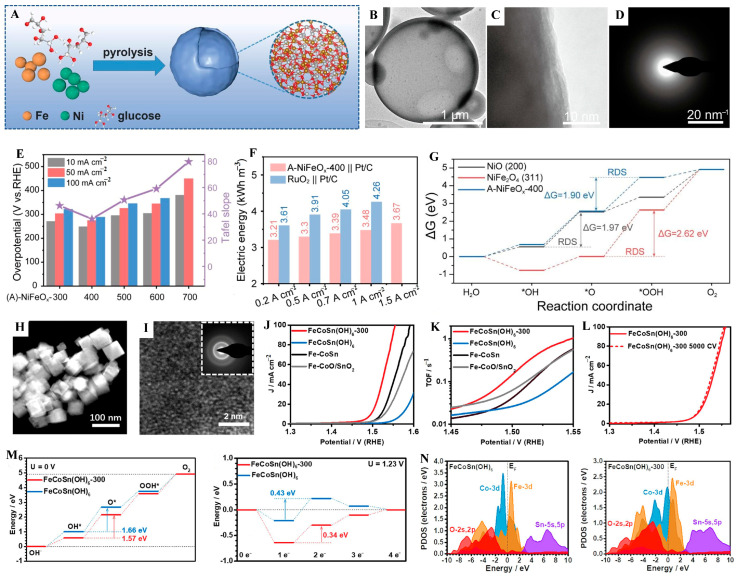
(**A**) Synthesis procedure of A-NiFeO_x_-400. (**B**,**C**) HRTEM image of the A-NiFeOx-400. (**D**) SAED image of the A-NiFeO_x_-400. (**E**) Overpotentials of different catalysts. (**F**) Comparison of electricity consumption to produce 1 cubic meter of H_2_. (**G**) Calculated free energy pathways for OER. Reproduced with permission from Ref. [76]. (**H**) HAADF-STEM image of CoSn(OH)_6_-300. (**I**) HRTEM images of CoSn(OH)_6_-300. (**J**) LSV curves and (**K**) TOF values collected at different potentials of FeCoSn(OH)_6_-300 and contrast materials. (**L**) OER polarization curves of FeCoSn(OH)_6_-300 after 5000 cycles. (**M**) The energetic trend of OER at U = 0 V and U = 1.23 V. (**N**) The PDOS of FeCoSn(OH)_6_ and FeCoSn(OH)_6_-300. Reproduced with permission from Ref. [77].

Furthermore, the amorphization of materials can induce localized charge redistribution around active sites, reducing the OER energy barrier through enhanced *OOH adsorption [75]. Guo et al. [76] used a spray pyrolysis approach and calcination at 400 °C to synthesize amorphous NiFe bimetallic oxide (A-NiFeO_x_-400) with tunable crystallinity, multilevel porosity, and atomic-scale compositional homogeneity (Figure 4A–D). The optimized A-NiFeO_x_-400 catalyst presented ultra-low overpotentials of 248, 274, and 288 mV at 10, 50, and 100 mA cm^−2^, respectively (Figure 4E), surpassing both crystalline analogues and commercial RuO_2_. Operando-attenuated total reflectance surface-enhanced infrared absorption spectroscopy indicated that the OER over A-NiFeO_x_-400 mainly follows the AEM under large current conditions. Density functional theory (DFT) calculations further demonstrated that structural amorphization triggers localized charge redistribution around Fe sites, which promotes *OOH adsorption and decreases the reaction energy barrier by 0.72 eV (Figure 4G). When integrated into an anion exchange membrane water electrolyzer (AEMWE), the A-NiFeO_x_-400 electrode delivered an exceptional industrial-level current density of 1 A cm^−2^ at 3.48 V (Figure 4F) while also demonstrating outstanding operational stability, retaining over 98.75% after 800 h at 1 A cm^−2^.

Liu et al. [77] showed a top-down strategy to prepare amorphous ternary metal oxides (Figure 4H,I). The experimental results prove that the amorphization process can significantly enhance the p-d coupling between Co and Fe sites (Figure 4N). This substantially reduces the energy barrier for the conversion of OH* to O*, which is exactly the rate-determining step of the OER (Figure 4M). Benefiting from the V_o_, disordered structure, and tunable electronic properties of amorphous oxides, the optimal FeCoSn(OH)_6_-300 exhibits a more superior electrocatalytic OER overpotential (266 mV at 10 mA cm^−2^) and turnover frequency (TOF) value than that of FeCoSn(OH)_6_ (Figure 4J–L).

#### 3.2.2. Reconstruction

Some metal oxides undergo dynamic and irreversible self-reconstruction in alkaline electrolytes and/or under anodic potentials, forming the true catalytic phase of transition metal oxyhydroxides (MOOH) [34]. Metal oxides based on the LOM reaction pathway often suffer surface reconstruction. During OER, the dynamic formation of V_o_ in the catalyst and the leaching of metal cations can both drive the transformation of the catalyst into an oxyhydroxide phase [78,79]. The MOOH obtained through this surface self-reconstruction process typically exhibits higher OER activity than pristine catalysts [80]. For example, in the Co_3_O_4_@(CoFeV)_3_O_4_ system during OER, the dissolution of V cations can lower the energy barrier for oxygen vacancy formation, thereby promoting material reconstruction. Importantly, such dynamic surface changes, including metal dissolution and oxygen vacancy formation, also enhance the stability of the electrocatalyst. As a result, the AEMWE system of Co_3_O_4_@(CoFeV)_3_O_4_ as the anode achieves an outstanding performance of 6.19 A cm^−2^ at 2.0 V in 1 M KOH solution, along with excellent durability for 96 h at 500 mA cm^−2^, approaching the requirements for industrial water electrolysis [81]. The self-reconstruction of catalysts during OER is spontaneous and often uncontrollable. Guan et al. [82] innovatively combined the operando strain with reconstruction effects to enhance the oxygen evolution reaction (OER) through the thermally self-assembling pristine Li_2_Co_2_O_4_ spinel into a composite structure of the layered LiCoO_2_ phase and the Co_3_O_4_ spinel. It has been shown that during OER, the layered Li_x_CoO_2_ phase exhibits significant tensile strain along the (003) plane, while the low-valent Co_3_O_4_ phase transforms into high-valent CoOOH_x_, achieving the synchronous occurrence of the operando strain and reconstruction. Further experimental and computational studies indicate that the E_OH_^−^ values for both the strained Li_x_CoO_2_ and the reconstructed CoOOH active phase are lower than those of the original phases, which is favorable for the adsorption of the OH^−^ reactant. Based on the synergistic effect of the operando strain in LiCoO_2_ and reconstruction in Co_3_O_4_ during OER, the material achieves excellent catalytic activity and stability.

### 3.3. Support Engineering

The inherently poor conductivity and intrinsic activity of TMOs significantly limit their electrocatalytic OER capability. Combining TMOs with supports featuring high conductivity or unique structures has emerged as an effective strategy. Dispersing TMO electrocatalysts within a support can increase the conductivity of the catalyst and/or exposed active surface area, thereby effectively improving the OER performance [83,84,85]. Furthermore, various supports often exhibit distinct characteristics, and their combination with catalysts can enhance physicochemical properties, such as conductivity, active surface area, stability, and electronic structure of the catalyst. This interaction between metal oxides and supports may significantly influence the OER performance of TMOs. Hence, three typical supports for TMO-based OER electrocatalysts will be discussed, including carbon materials, MXene, and graphitic carbon nitride materials (g-C_3_N_4_).

#### 3.3.1. Carbon Support

Among carbon materials, graphene, carbon nanotubes, and carbon nanofibers serve as excellent conductive supports. The carbon-based composite materials constructed from them have been widely used in the field of electrochemistry. Due to the confinement effect of carbon materials, transition metal oxide particles with smaller sizes and uniform distribution on the carbon support can be obtained, thereby increasing the exposed active surface area of electrocatalysts [86,87].

**Figure 5:**
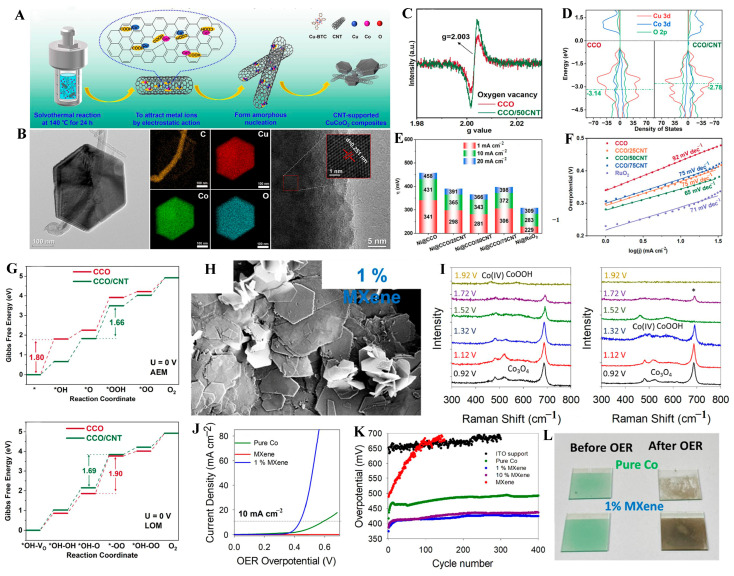
(**A**) Synthesis procedure of the CNT-supported CuCoO_2_ nanosheet. (**B**) The TEM image, the elemental mapping, and the HRTEM image of CCO/50CNT. (**C**) EPR spectra and the (**D**) PDOS of CCO and CCO/CNT. (**E**) Overpotential comparison and (**F**) Tafel slope of CCO/50CNT and contrast materials. (**G**) The Gibbs free energy diagrams of CCO and CCO/CNT for AEM and LOM pathways. Reproduced with permission from Ref. [89]. (**H**) SEM image of 1% MXene. (**I**) in situ Raman spectroscopy of Co_3_O_4_ and 1% MXene. * represents Co_3_O_4_. (**J**) LSV curves. (**K**) Effect of multi-cycling into and out of the OER region for as-synthesized materials. (**L**) Photo of the as-synthesized catalysts before and after OER. Reproduced with permission from Ref. [97].

Hanan et al. [88] synthesized a Co_2_FeO_4_/rGO composite (CFG-10) via a simultaneous hydrothermal and reduction method. Owing to the synergistic effects between Co_2_FeO_4_ and rGO, the resulting catalyst revealed a large specific surface area, high electrical conductivity, optimized exposed active sites, multiple charge transport pathways, and V_o_ at the surface of CFG-10. As a result, the CFG-10 catalyst demonstrated a low overpotential of 240 mV for the OER at 10 mA cm^−2^, along with remarkable stability over 48 h. CNTs have acquired tremendous attention from numerous researchers, owing to high electrical conductivity, excellent stability, and large specific surface area. CNTs can not only provide adsorption sites for metal ions and suppress catalyst aggregation, but also facilitate rapid electron transfer pathways. Ma et al. [89] successfully synthesized CuCoO_2_ nanosheets supported on CNTs (CCO/50CNT) via a one-step hydrothermal method (Figure 5A). The interaction between the carbon nanotubes and the CuCoO_2_ reduced the thickness of the CuCoO_2_ to 25 nm (Figure 5B) and increased the V_o_ concentration in the material (Figure 5C). The addition of CNT shifted the d-band center (εd) closer to the Fermi level, which facilitated the Eads of O-containing intermediates during the OER (Figure 5D). The CCO/50CNT showed an overpotential of only 343 mV at 10 mA cm^−2^, along with a Tafel slope of 65 mV dec^−1^ (Figure 5E,F). DFT calculations showed that the Gibbs free energy (ΔG = 1.69) of the rate-determining step (RDS) of the LOM was nearly equal to that of the RDS of the AEM (1.66) for CCO/CNT, which indicates the concurrent operation of the AEM and the LOM on the catalyst surface during the OER (Figure 5G).

Electrospinning technology enables the uniform embedding of metal compounds within carbon nanofibers (CNFs). The carbon matrix retained within the catalyst not only enhances the electrical conductivity of the metal oxides but also prevents nanoparticle aggregation. For instance, CoFe_2_O_4_ nanoparticles were uniformly embedded in one-dimensional carbon nanofibers via electrospinning to form CFO@CNFs. It demonstrated that the synergistic effect between the redox activity of CoFe_2_O_4_ and the large surface area, along with the excellent conductivity of CNFs, significantly enhanced the OER performance. The CFO@CNFs-800 sample exhibited a reduced overpotential of 300 mV at 10 mA cm^−2^, as well as outstanding long-term stability [90]. Similarly, MIL-53(Fe) and Ni(NO_3_)_2_ were incorporated into carbon nanofibers (CNFs) through electrospinning. After carbonization, Fe_2_O_3_ and NiFe_2_O_4_ nanoparticles were tightly embedded within the CNF framework, forming Fe_2_O_3_/NiFe_2_O_4_@CNFs. The obtained catalyst possessed a rough and porous surface and abundant oxygen vacancies, which facilitated efficient mass transport and exposed more active sites for energy conversion. For the OER, the Fe_2_O_3_/NiFe_2_O_4_@CNFs demonstrated excellent electrocatalytic activity. Moreover, after stability testing, the average size of the Fe_2_O_3_/NiFe_2_O_4_ nanoparticles remained well-preserved without significant aggregation or expansion [91].

#### 3.3.2. MXene Support

As a class of 2D transition metal carbides, nitrides, or carbonitrides, MXene exhibits high electrical conductivity, superior mechanical flexibility, and good hydrophilicity [92,93]. The presence of surface functional groups such as -O, -OH, and -F enables MXene to interact effectively with polar metal cations or polar oxides [94,95]. This interaction contributes to enhanced electrical conductivity and active sites of electrocatalysts. Wang et al. [96] assembled Co_3_O_4_ quantum dots on Ti_3_C_2_T_x_ MXene nanosheets via van der Waals interactions. The resulting Co_3_O_4_ quantum dots/MXene composite exhibited distinct advantages in OER performance through synergistic effects. The flexible and conductive Ti_3_C_2_T_x_ nanosheets not only facilitated rapid electron and ion transport but also suppressed the aggregation of Co_3_O_4_ quantum dots. Moreover, the Co_3_O_4_ nanoparticles acted as spacers that prevented the restacking of MXene nanosheets and sustained the integrity of active regions. As a result, the Co_3_O_4_ quantum dots/MXene composite demonstrated outstanding oxygen evolution performance. In particular, Tyndall et al. [97] carried out an in-depth investigation into the interaction between MXene and Co_3_O_4_ (Figure 5H). Through in situ Raman spectroscopy analysis, it was confirmed that the enhanced conductivity of the composite matrix enabled cobalt species to reach higher oxidation states at lower applied potentials (Figure 5I). The resulting high-oxidation-state cobalt (Co^4+^) exhibited greater reactivity on the catalyst surface while requiring a lower overpotential, compared to pristine Co_3_O_4_ (Figure 5J). Furthermore, the catalyst also exhibited enhanced mechanical robustness, as shown by the development of a durable film after electrochemical cycling (Figure 5K,L).

A report developed a simple hydrothermal method to decorate NiFe_2_O_4_ nanoparticles onto Ti_3_C_2_ MXene nanosheets. The NiFe_2_O_4_/Ti_3_C_2_ composite exhibited a low overpotential of 266 mV at 10 mA cm^−2^ and a small Tafel slope of 73.6 mV/dec for OER. The excellent electrocatalytic property of the NiFe_2_O_4_/Ti_3_C_2_ was attributed to the constructed nanoparticle/nanosheet interface synergistic effects and the high metallic conductivity of the Ti_3_C_2_ nanosheets. The nanoparticle/sheet interface promoted greater exposure of active sites, thereby enhancing the OER performance. DFT revealed that the interaction between NiFe_2_O_4_ and Ti_3_C_2_ involved van der Waals forces and chemical interactions, such as charge transfer from the NiFe_2_O_4_ to Ti_3_C_2_, demonstrating the strong synergy between NiFe_2_O_4_ and Ti_3_C_2_ [98]. Another report pointed out that the synergy between MXene nanosheets and NiFe_2_O_4_ nanoparticles optimized the E_ads_ of O-containing intermediates, thereby improving the overall catalytic efficiency [99].

#### 3.3.3. Graphitic C-N Support

g-C_3_N_4_ has garnered significant interest due to its chemical/thermal stability, abundant active sites, favorable electron mobility, and low cost. Nevertheless, its electrocatalytic performance is limited by inefficient reactive sites and poor electrical conductivity [100,101]. Recent studies have shown that chemical interactions between the surface/interface atoms of g-C_3_N_4_ and the suitable metal oxides can enhance the electrocatalytic activity, thereby promoting charge transfer and OER kinetics [102,103]. For example, Vignesh et al. [104] constructed a tightly integrated two-dimensional composite electrocatalyst by combining α-Fe_2_O_3_ with Co_3_O_4_/g-C_3_N_4_ nanostructures to modulate the electronic structure and further enhance the electrocatalytic OER activity. The persistent interfacial contact and synergistic coupling between α-Fe_2_O_3_ and the Co_3_O_4_/g-C_3_N_4_ nanostructure provided a large specific surface area and numerous active sites with enhanced electron transfer capability, leading to significantly accelerated electrocatalytic kinetics.

Sundararaj et al. [105] synthesized NiSnO_3_ nanospheres anchored on ultrathin g-CN nanosheets using a facile hydrothermal method. The formation of this nanohybrid structure was confirmed by XPS analysis, which revealed the presence of interfacial Ni–N bonds. The strong affinity between the NiSnO_3_ nanospheres and g-CN nanosheets facilitated electron transfer, thereby improving electrocatalytic performance. The high specific surface area of the resulting nanohybrid catalyst strongly suggested the presence of enhanced OER active sites. Compared to NiSnO_3_ (260 mV) and g-CN (330 mV), the NiSnO_3_/g-CN exhibited a lower overpotential (240 mV), along with electrochemical stability.

Similarly, a 3D flower-like composite catalyst composed of NiCo_2_O_4_ nanosheets and N-doped carbon derived from g-C_3_N_4_ was developed. The resulting three-dimensional structure facilitated the transport of species (oxygen and ions), which are beneficial for OER performance. In addition, the modified ultrathin C-N compound enhanced the electrical conductivity of NiCo_2_O_4_. This hybrid catalyst exhibited outstanding oxygen evolution reaction performance, demonstrating the feasibility of using two-dimensional layered C-N materials to improve electrochemical properties [106].

### 3.4. Heteroatom Doping

The energy barrier of the OER is primarily dictated by the binding strength between oxygen-containing intermediates and the active sites of catalyst. This is a fundamental aspect governed by the electronic structure of the d-orbitals, which directly regulates intermediate adsorption/desorption [107,108]. It has been demonstrated that doping with foreign cations and/or anions can efficiently modulate the electronic structure (e.g., d-band center and orbital hybridization), optimize geometric structure (e.g., oxygen vacancies and lattice strain), and alter the reaction pathway (e.g., AEM and LOM), thereby influencing the OER mechanism [109,110]. Specifically, cation doping often activates the lattice oxygen mechanism (LOM) through structural reconstruction, whereas anion doping promotes LOM via orbital engineering. Consequently, regulating the type and ratio of cation and anion doping offers a viable route to enhancing the ORR performance of electrocatalysts.

#### 3.4.1. Cationic Doping

IrO_2_ and RuO_2_, as noble metal electrocatalysts, are highly favored for their exceptional activity and stability in the OER. However, the high cost impedes the large-scale application of noble metals. Interestingly, it has been shown that incorporating small amounts (0.5–3%) of precious metals like Ir or Ru into transition metal oxides can significantly reduce the usage of these precious metals while still maintaining excellent OER performance [111,112]. Ir-doped Co_3_O_4_ catalysts can induce lattice distortion and enhance the number of active sites in Co_3_O_4_, thereby increasing the OER catalytic property. As a consequence, it exhibits an overpotential of 225 mV at 10 mA cm^−2^ and a Tafel slope of 64.1 mV dec^−1^, both of which are lower than those of Co_3_O_4_ and IrO_2_, and it also demonstrates excellent stability [113]. Tian et al. [114] prepared Ru-doped Co_3_O_4_ supported on carbon cloth (RCO-V_o_@CC) through high-temperature calcination. The oxygen vacancies (V_o_) generated by Ru doping effectively modulated the electronic structure of Co_3_O_4_, leading to strong interfacial interactions between Ru and Co_3_O_4_. Theoretical calculations indicated that Ru doping improved the metallic character of Co_3_O_4_. The hybridization of Ru and Co orbitals induced strong electronic interactions, resulting in an upward shift of the d-band center. This strengthened the interaction between the catalyst and adsorbates, reduced the reaction energy barrier, and accelerated the catalytic kinetics. Consequently, the OER performance was significantly improved, achieving an overpotential of 253 mV at 10 mA cm^−2^.

**Figure 6:**
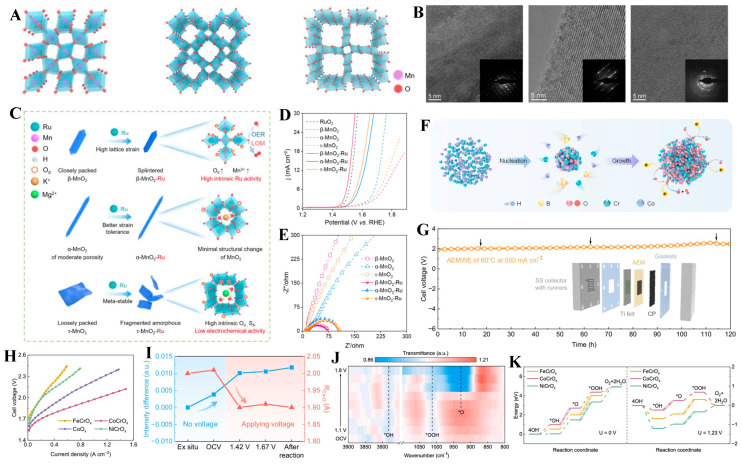
(**A**) Schematic lattice structure; (**B**) TEM images of β-MnO_2_, α-MnO_2_, and τ-MnO_2_, sequentially. (**C**) Schematic illustration of the strain-induced structural changes for MnO_2_ of various polymorphs. (**D**) LSV curves and (**E**) Nyquist plots of β-MnO_2_-Ru and contrast materials. Reproduced with permission from Ref. [110]. (**F**) Illustration of the preparation procedure of CoCrOx. (**G**) Chronopotentiometry curve of CoCrO_x_ in the AEMWEs. (**H**) I-V curves of AEMWEs, with the prepared catalysts as anodes. (**I**) Relationship between intensity differences in in situ vtc-XES spectra and Co-O bond lengths during the OER process. (**J**) In situ SRIR measurements under various potentials for CoCrO_x_ during the OER process. (**K**) The energetic trend of OER at U = 0 V and U = 1.23 V. Reproduced with permission from Ref. [118].

Qin et al. [110] discovered that Ru doping into different crystalline types of MnO_2_ induced distinct lattice strains and morphological changes (Figure 6A,B). α-MnO_2_ exhibited minimal structural and morphological alterations after Ru doping, owing to its relatively stable porous structure that can accommodate heteroatom incorporation. In contrast, Ru-doped β-MnO_2_ underwent crystal fracture in its closely packed structure, leading to significant changes in surface area, oxygen vacancies, and manganese valence states. τ-MnO_2_ proved less stable upon Ru doping and then transformed into an amorphous phase (Figure 6C). For OER performance, Ru-doped β-MnO_2_ achieved the lowest overpotential (278 mV) at 10 mA cm^−2^ (Figure 6D) and the highest conductivity (Figure 6E). Therefore, Ru incorporation not only increases the number of active sites on the MnO_2_ surface but also modifies the crystal structure of MnO_2_, thereby reversibly modulating its catalytic activity.

Non-noble metals, which are abundant, can be utilized for doping to reduce the high costs associated with noble metals. Moreover, they effectively optimize the electronic structure of TMOs and enhance the electrocatalytic OER performance [115,116,117]. Li et al. [118] prepared a series of Cr-doped amorphous metal oxides (CoCrO_x_, NiCrO_x_, FeCrO_x_) via the NaBH_4_ reduction method in an aqueous solution (Figure 6F). In the AEMWE system, the CoCrO_x_ catalyst showed a high current density of 1.5 A cm^−2^ at 2.1 V and maintained it for over 120 h, with attenuation being less than 4.9 mV h^−1^ (Figure 6G,H). Cr-doping reduced the average valence state of Co and the length of Co-O bonds. During the OER, the shorter Co-O bonds in CoCrO_x_ optimized the binding energy for oxygen intermediate adsorption, enabling *O to readily adsorb onto Co sites (Figure 6I–K). This led to an increase in the number of Co ligands and changes in the Co valence state, thereby promoting the oxygen ionization reaction process. In catalytic systems, active sites are either metal sites or lattice oxygen. Therefore, if both metal sites and lattice oxygen can be simultaneously activated to form redox pairs with low energy barriers, thereby providing diversified active sites, it holds promise for accelerating the rate of the OER. Based on this, Wang et al. [119] designed a catalyst supported on Ir and Ni co-doped Co_3_O_4_ (Ir/Ni-Co_3_O_4_). In situ spectroscopy, chemical probe experiments, and isotope labeling indicated that both metal sites and lattice oxygen are synchronously activated to participate in the OER. DFT calculations revealed that Co sites contribute to oxygen electrocatalytic activity by accommodating OER intermediates with a low energy barrier of 1.69 eV. Ni and Ir atoms synergistically elevated the energy position of the O-band center. Consequently, the lattice oxygen bridging Ni and Ir atoms was activated through coupling with the adsorbed oxygen on Ir sites, thereby facilitating O-O bond formation. Benefiting from the synergistic effect between metal sites and lattice oxygen, Ir/Ni-Co_3_O_4_ achieved remarkably low OER overpotentials of 177, 218, and 263 mV at current densities of 10, 100, and 500 mA cm^−2^, respectively, indicating outstanding OER performance.

#### 3.4.2. Anionic Doping

**Figure 7:**
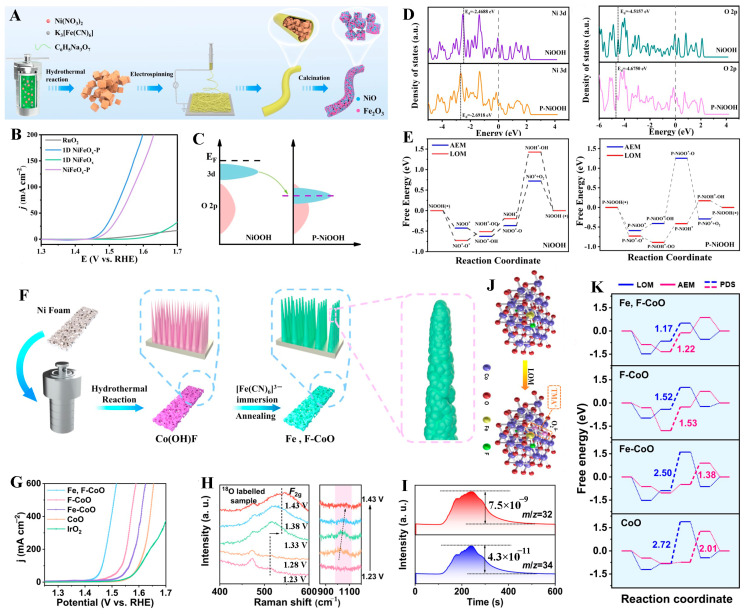
(**A**) Synthesis scheme for 1D NiFeOx. (**B**) LSV curves of as-prepared catalysts. (**C**) Schematic band structures of P-NiOOH and NiOOH. (**D**) Density of states of Ni 3d of P-NiOOH and NiOOH. (**E**) Free energy of OER steps via AEM and LOM mechanisms on P-NiOOH. Reproduced with permission from Ref. [121]. (**F**) Schematic illustration of the synthetic procedure of the Fe, F-CoO NNAs catalyst. (**G**) LSV curves of Fe, F-CoO NNAs and contrast materials. (**H**) In situ Raman spectra of Fe, F-Co^18^O NNAs. (**I**) On-line DEMS spectra of Fe, F-Co^18^O NNAs. (**J**) Schematic illustration of the chemical recognition of negatively charged peroxo/superoxo-like species in the LOM. (**K**) Gibbs free energy of the Fe, F-CoO and contrast materials via AEM and LOM pathways. Reproduced with permission from Ref. [124].

Anions with lower electronegativity exhibit higher covalency in their bonds with metals. This results in weaker electron attraction, which facilitates charge transport and modulates the adsorption energy of reaction intermediates during the OER [120]. Yu et al. [35] prepared an N-doped two-dimensional CoO catalyst via liquid-phase and chemical vapor deposition. From the testing result, it can observe that both CoO and CoN catalysts exhibited a gradually intensifying spectral band around 950 cm^−1^ after reaching the oxygen evolution potential (≥1.3 V), corresponding to the OOH* intermediate. By contrast, no such band appeared on the N-doped CoO catalyst. N-doped CoO exhibited a distinct absorption band at 1065 cm^−1^ upon reaching potentials ≥1.3 V, assigned to the O-O* species, a key intermediate preceding oxygen release. These results reveal that the incorporation of nitrogen alters the OER mechanism from the adsorbate AEM to the LOM. Furthermore, it has been revealed that electron transfer proceeds from oxygen non-bonding states rather than from the Co-O bonds, thereby preserving the integrity of the Co-O framework during oxygen evolution and ensuring exceptional OER stability. In a practical AEMWE, the catalyst at the anode exhibited a high current density of 1000 mA cm^−2^ at 1.78 V and no voltage increase over 300 h at 1 A cm^−2^. Li et al. [121] prepared one-dimensional P-doped NiFeO_x_ catalysts (NiFeO_x_-P) through electrospinning and high-temperature phosphidation (Figure 7A). DFT calculations elucidated that P-doping induced a downshift in the Ni 3d band center alongside a slight upward shift in the O 2p band center, leading to an enhanced overlap between the Ni 3d and O 2p orbitals (Figure 7C,D). This overlap significantly increased the M-O covalency, thereby activating the LOM. Furthermore, the free energy diagrams of NiOOH revealed that the energy barrier for the AEM on the undoped NiOOH was much lower than that for LOM, indicating that the NiOOH follows the AEM pathway (Figure 7E). In contrast, for phosphorus-doped NiOOH, the energy barrier for AEM was substantially higher than that for LOM, confirming that the reaction pathway shifted to LOM upon doping. The enhanced M-O covalency promoted the transition of the OER pathway from AEM to LOM, thereby breaking the relationship between the E_ads_ of *OH and *OOH intermediates in AEM. As a result, the obtained NiFeO_x_-P exhibited a low overpotential of 237 mV at 10 mA cm^−2^, along with durable stability (Figure 7B).

The dual-doping strategy involving both cations and anions has attracted significant attention, as it simultaneously enables lattice oxygen activation and enhances the local electric field to facilitate the OER [25,122,123]. For instance, Ye et al. [124] synthesized iron and fluorine co-doped cobalt oxide nano-nail arrays (Fe, F-CoO NNAs) via a hydrothermal reaction (Figure 7F). The material exhibited ultra-low overpotentials of 169 mV, 234 mV, and 277 mV at 10 mA cm^−2^, 100 mA cm^−2^, and 500 mA cm^−2^, respectively (Figure 7G). Experimental results and theoretical calculations confirmed that the dual doping of Fe and F synergistically upshifted the Co d-band center and the O 2p band near the Fermi level. This enhancement increased the M-O covalency, activated lattice oxygen, facilitated electron transfer, and thereby triggered the LOM pathway (Figure 7H–J). In addition, Fe doping markedly enhanced the local charge density by the coupling of the sharp-tip enhancement effect with the proximity effect, which attracted more OH^−^, optimized the reaction energy barrier of the LOM pathway, and facilitated O_2_ release (Figure 7K). As a result, both the thermodynamic and kinetic energy barriers of the OER were reduced, leading to excellent electrocatalytic performance.

### 3.5. Heterostructure Construction

The construction of heterostructures serves as an effective strategy for electronic structure modulation. The built-in electric field at the heterointerface promotes charge transfer and electron rearrangement, thereby optimizing active sites, while the synergistic effects between different active sites enhance the kinetics of the OER [125,126,127]. Meanwhile, the heterostructure effectively suppresses the degeneration of active sites, thereby improving the satisfactory durability [128,129].

**Figure 8:**
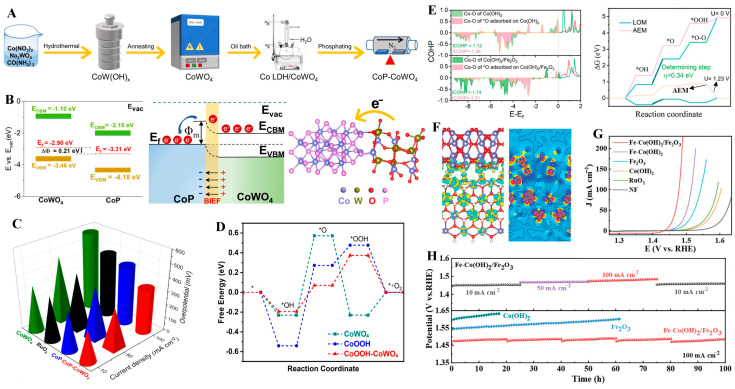
(**A**) Schematic outlining the synthetic route to the CoP-CoWO_4_ heterostructure. (**B**) Energy band diagram of CoWO_4_ and CoP, schematic diagram of band structures, and schematic of charge migration. (**C**) Overpotentials. (**D**) Gibbs free energy diagram at U = 1.23 V. Reproduced with permission from Ref. [131]. (**E**) The integrated crystal orbital Hamilton population and Gibbs free energy diagram of OER steps for Fe-Co(OH)_2_/Co(OH)_2_. (**F**) Differential charge density. (**G**) LSV curves of as-prepared catalysts. (**H**) The stability of Fe-Co(OH)_2_/Fe_2_O_3_. Reproduced with permission from Ref. [132].

Recently, Song et al. [130] synthesized NiO/CoFe_2_O_4_ heterostructures through the hydrothermal method, followed by high-temperature calcination. Combined XPS and work function analyses revealed that the heterostructure formation triggered electronic modulation at the interface, enhancing the E_ads_ of OH^−^ and deprotonation capabilities, thereby facilitating the adsorption and decomposition of oxygen-containing intermediates. As a result, the NiO/CoFe_2_O_4_ heterostructure exhibited an overpotential of 270 mV at 10 mA cm^−2^ and remained stable for over 30 h. Yan et al. [131] fabricated a CoP-CoWO_4_ heterostructure via the precise management of the synthesis process (Figure 8A). Owing to the difference in work function between CoP and CoWO_4_, the electrons of the heterointerface were driven to transfer from CoWO_4_ to CoP, which induced an asymmetric charge distribution at the interface and further enabled the spontaneous formation of a built-in electric field (BIEF) (Figure 8B). The special electronic structure can restructure the adsorption/desorption kinetics of oxygen intermediates (Figure 8D), resulting in a remarkable enhancement of the OER property. Specifically, CoP-CoWO_4_ achieved a low overpotential of only 241 mV at a current density of 10 mA·cm^−2^, outperforming the CoWO_4_ (347 mV) and CoP (300 mV) catalysts (Figure 8C). Xin et al. [132] reported a Fe-Co(OH)_2_/Fe_2_O_3_ heterostructure via a one-step electrodeposition method. Density functional theory (DFT) calculations and chemical probe experiments demonstrated that, benefiting from the synergistic effect between Fe-Co(OH)_2_ and Fe_2_O_3_, the two components followed the AEM and LOM mechanisms, respectively (Figure 8E). This allowed the metal active sites to exhibit stronger adsorption capacity for oxygen-containing intermediates, rapidly complete the deprotonation process, and trigger the O-O bond formation step at lattice oxygen sites. Meanwhile, the formation of a BIEF derived from the p-n structure between Fe-Co(OH)_2_ and Fe_2_O_3_ modulated the electronic configuration (Figure 8F), which further enhanced the activity and stability of the catalyst. Consequently, the Fe-Co(OH)_2_/Fe_2_O_3_ heterostructure delivered an overpotential of only 219 mV at current densities of 10 mA·cm^−2^ (Figure 8G) and long-term stability for 100 h under a current density of 100 mA·cm^−2^ (Figure 8H).

The latest research has confirmed that combining magnetic fields with electrochemistry presents a novel and promising strategy for enhancing electrochemical reactions. Based on this, Zhang et al. [133] integrated NiFe-LDH nanosheets with Co_3_O_4_ nanowires constructed on ferromagnetic nickel foam (NiFe-LDH/Co_3_O_4_/NF) to synergistically improve OER performance through internal p-n heterojunction electronic structure modulation and external magnetic field effects. This p-n heterojunction induced charge redistribution at the heterogeneous interface to achieve Fermi-level alignment, thereby reducing the ΔG of *OOH and enhancing the inherent activity and conductivity of the catalyst. Consequently, NiFe-LDH/Co_3_O_4_/NF presented an excellent OER property, with a low overpotential of 274 mV at 50 mA cm^−2^ and long-term stability over 90 h. Furthermore, the ferromagnetic Ni foam acted as a magnetic core, inducing an exchange bias effect between the magnetic substrate and active species under the magnetic field, which reduced magnetoresistance and weakened spin electron scattering, thereby accelerating electron transport during the OER. Under a magnetic field of 10,000 G at 50 mA cm^−2^, the OER overpotential was further reduced by 25 mV. These findings demonstrate that constructing p-n heterojunctions on magnetic substrates may be an effective strategy for developing high-efficiency magnetization effect catalysts. Lastly, a summary table comparing the key OER performance of the discussed catalysts is provided in Table 1.

## 4. Conclusions and Outlook

TMOs are generally regarded as ideal materials for replacing precious metal catalysts in OER due to their low cost, abundant resources, and structural stability and tunability. However, the inherent drawbacks of transition metal oxides, such as poor electrical conductivity and limited catalytic activity, hinder their practical application. Current research focuses on optimizing the electronic structure and physicochemical environment of TMOs through various strategies to enhance OER performance. Hence, we summarized recent optimization strategies and corresponding mechanisms for improving the OER performance of TMO electrocatalysts. These strategies, including morphology control, support engineering, heteroatom doping, and heterostructure construction, have significantly enhanced the electrocatalytic performance of transition metal oxides. Morphology control involves shaping catalysts into two-dimensional ultrathin nanosheets, hollow cage-like structures, etc., which can maximize the exposure of active sites, shorten ion/electron transport paths, and facilitate the exposure of high-index crystal planes, thereby improving mass transport efficiency. Support engineering entails highly dispersing the active oxide components onto conductive or structurally unique supports, which not only prevents particle agglomeration and enhances stability but also further improves electron conduction and catalytic performance through interactions between the support and the active components. Heteroatom doping involves introducing heteroatoms (elemental doping) or constructing bimetallic centers to effectively modulate the electronic state of the metal centers, optimizing their adsorption/desorption energy for oxygen intermediates and thereby significantly enhancing intrinsic catalytic activity. The synergistic effect between bimetallic centers is key to performance improvement. Heterostructure construction, which involves creating heterostructure interfaces or introducing defects, such as oxygen vacancies, into the lattice, can effectively regulate charge distribution, accelerate interfacial charge transfer, and expose more highly active sites, thereby reducing the reaction energy barrier.

Each of the above enhancement strategies has its own strengths and weaknesses. For TMOs with extremely poor conductivity, improving conductivity via support engineering is the primary and most significant strategy. When the basic conductivity is addressed, electronic structure regulation (via doping, heterojunction construction, or crystal facet control) becomes the key to further enhancing intrinsic activity. In contrast, macroscopic morphology engineering mainly functions in optimizing mass transfer and increasing the number of active sites, and its effect has an upper limit. Currently, the elaboration of mechanisms such as the crystal facet effect and heterojunction synergy relies on multiple characterizations and calculations, with relatively sufficient evidence. However, direct evidence is still lacking for the formation of real active phases via dynamic reconstruction and the LOM pathway.

Despite significant progress, the transition of TMO catalysts from the laboratory to practical applications still faces numerous challenges, which also represent key directions for future research, as shown as follows:

(1) Precise synthesis and structural control: Achieving precise and controllable synthesis of composition, crystal phase, size, morphology, and defect types remains highly challenging. Particularly for multi-metal oxides, the demanding synthesis conditions increase the complexity and unpredictability of preparation. Future efforts require the development of more scientific, precise, and scalable synthetic methods. These methods should be environmentally friendly, possess high atom economy, and enable the direct construction of stable catalyst structures on current collectors to ensure their practical application potential.
(2) Active site identification and mechanism elucidation: The dynamic reconstruction of the catalyst surface during the OER process makes identifying the true active sites and stable phases extremely challenging. In the future, the development of time-resolved rapid in situ characterization technologies (such as time-resolved X-ray absorption spectroscopy, fast-scanning electrochemical microscopy, in situ transient spectroscopy, etc.) will be crucial. These technologies can track the structural evolution of active sites and the formation and transformation of intermediates during the catalytic process on the millisecond or even microsecond scale, thus more accurately revealing the real-time reaction pathway and deactivation mechanism of OER.
(3) Stability and degradation mechanisms: The dissolution kinetics and long-term durability of catalysts remain focal points of debate. Under the high anodic potentials of OER, oxide dissolution is a dynamic process whose mechanisms are not yet fully understood. There is a need to develop characterization protocols capable of tracking changes in catalyst structure, valence state, and composition over extended periods to systematically study degradation mechanisms. Strategies such as introducing vacancy defects to enhance structural stability and corrosion resistance are effective pathways for improving longevity.
(4) Multifunctional application and broad-pH-range adaptability: Currently, high-performance catalysts are mostly confined to alkaline environments, with severely inadequate activity and stability in acidic or neutral media, limiting their application scope. Developing catalysts that exhibit both high activity and high stability across a broad pH range, especially under acidic conditions, is a critical future direction. Simultaneously, designing bifunctional or even trifunctional catalysts that couple OER with the HER and the oxygen reduction reaction (ORR) is crucial for realizing next-generation energy conversion devices, such as overall water splitting and metal–air batteries.
(5) Artificial intelligence and machine learning technologies: AI can be applied to high-throughput screening to predict the components of TMOs with desirable electronic structures, stability, and activity; identify key descriptors from multi-dimensional data and establish a “structure–performance” relationship model; and guide the intelligent regulation of experimental conditions (such as temperature, precursor ratio, atmosphere, etc.), so as to realize the precise and controllable preparation of catalysts. In the future, the paradigm shift from “trial-and-error” research to “rational design-experimental validation” will be realized, which will greatly accelerate the development process of TMO catalysts and promote their translation from laboratory to industrial applications.

TMOs hold promising prospects as OER catalysts. Future research should move beyond the optimization of single strategies and shift towards a design philosophy emphasizing the synergistic integration of multiple strategies, deeply combining composition modulation, support engineering, and morphology control. Concurrently, leveraging advanced characterization and theoretical simulations to deeply unravel catalytic mechanisms and degradation pathways and committing to the development of broad-pH-range, multifunctional catalyst systems will be key to advancing these high-performance, low-cost catalysts towards industrial application.

## Figures and Tables

**Table 1:** List of the discussed catalysts on mechanism transformation and main research contents.

Catalyst	Modulation	Electrolyte	Mechanism	Overpotential	Stability	Ref.
LaCoO_3_ (100) film	Crystal orientation	1 M KOH	AEM	470 mV at 6.58 A g^−1^	20,000 s at 6.58 A g^−1^	[64]
Vo-Co_3_O_4_ HNCs	Hollow structures	0.50 M H_2_SO_3_	LOM	265 mV at 10 mA cm^−2^	130 h at 20 mAcm^−2^	[70]
A-NiFeOx-400	Amorphization	1 M KOH	AEM	248 mV at 10 mA cm^−2^	16 h at 10 mA cm^−2^	[76]
FeCoSn(OH)_6_-300	Amorphization	1 M KOH	AEM	266 mV at 10 mA cm^−2^	200 h at 100 mA cm^−2^	[77]
CCO/50CNT	Carbon Support	1 M KOH	AEM and LOM	343 mV at 10 mA cm^−2^	18 h at 10 mA cm^−2^	[89]
Ti_3_C_2_T_x_ sheets/NiFe_2_O_4_	MXene Support	1 M KOH		181 mV at 10 mA cm^−2^		[99]
*β*-MnO_2_-Ru	Cationic Doping	1 M KOH	LOM	278 mV at 10 mA cm^−2^	50 h at 10 mA cm^−2^	[110]
CoCrO_x_	Cationic Doping	1 M KOH	AEM	268 mV at 10 mA cm^−2^	120 h at 500 mA cm^−2^	[118]
NiFeO_x_-P	Anionic Doping	1 M KOH	LOM	237 mV at 10 mA cm^−2^	120 h at 70 mA cm^−2^	[121]
Fe, F-CoO NNAs	Doping	1 M KOH	LOM	169 mV at 10 mA cm^−2^	300 h at 500 mA cm^−2^	[124]
Fe-Co(OH)_2_/Fe_2_O_3_	Heterostructure	1 M KOH	AEM and LOM	219 mV at 10 mA cm^−2^	100 h at 100 mA cm^−2^	[132]
NiFe-LDH/Co_3_O_4_/NF	Heterostructure	1 M KOH	AEM	274 mV at 50 mA cm^−2^	48 h at 50 mA cm^−2^	[133]

## Data Availability

No new data were created or analyzed in this study. Data sharing is not applicable to this article.
